# Substance P analogs devoid of key residues fail to activate human mast cells *via* MRGPRX2

**DOI:** 10.3389/fimmu.2023.1155740

**Published:** 2023-05-09

**Authors:** Shammy Raj, Stepan Hlushak, Narcy Arizmendi, Andriy Kovalenko, Marianna Kulka

**Affiliations:** ^1^ Nanotechnology Research Centre, National Research Council Canada, Edmonton, AB, Canada; ^2^ Department of Mechanical Engineering, University of Alberta, Edmonton, AB, Canada; ^3^ Department of Medical Microbiology and Immunology, Katz Group Centre, University of Alberta, Edmonton, AB, Canada

**Keywords:** MRGPRX2, mast cells, substance P, amino acid residues, ligand-receptor interactions

## Abstract

Mast cells play an important role in disease pathogenesis by secreting immunomodulatory molecules. Mast cells are primarily activated by the crosslinking of their high affinity IgE receptors (FcεRI) by antigen bound immunoglobulin (Ig)E antibody complexes. However, mast cells can also be activated by the mas related G protein-coupled receptor X2 (MRGPRX2), in response to a range of cationic secretagogues, such as substance P (SP), which is associated with pseudo-allergic reactions. We have previously reported that the *in vitro* activation of mouse mast cells by basic secretagogues is mediated by the mouse orthologue of the human MRGPRX2, MRGPRB2. To further elucidate the mechanism of MRGPRX2 activation, we studied the time-dependent internalization of MRGPRX2 by human mast cells (LAD2) upon stimulation with the neuropeptide SP. In addition, we performed computational studies to identify the intermolecular forces that facilitate ligand-MRGPRX2 interaction using SP. The computational predictions were tested experimentally by activating LAD2 with SP analogs, which were missing key amino acid residues. Our data suggest that mast cell activation by SP causes internalization of MRGPRX2 within 1 min of stimulation. Hydrogen bonds (h-bonds) and salt bridges govern the biding of SP to MRGPRX2. Arg1 and Lys3 in SP are key residues that are involved in both h-bonding and salt bridge formations with Glu164 and Asp184 of MRGPRX2, respectively. In accordance, SP analogs devoid of key residues (SP1 and SP2) failed to activate MRGPRX2 degranulation. However, both SP1 and SP2 caused a comparable release of chemokine CCL2. Further, SP analogs SP1, SP2 and SP4 did not activate tumor necrosis factor (TNF) production. We further show that SP1 and SP2 limit the activity of SP on mast cells. The results provide important mechanistic insight into the events that result in mast cell activation through MRGPRX2 and highlight the important physiochemical characteristics of a peptide ligand that facilitates ligand-MRGPRX2 interactions. The results are important in understanding activation through MRGPRX2, and the intermolecular forces that govern ligand-MRGPRX2 interaction. The elucidation of important physiochemical properties within a ligand that are needed for receptor interaction will aid in designing novel therapeutics and antagonists for MRGPRX2.

## Introduction

Mast cells (MCs) are sentinel and tissue-resident immune cells that rapidly release a diverse set of immune mediators upon activation (1–3). MCs are important immunomodulatory cells, which are involved in both the homeostatic process as well as in the pathogenic events in several diseases including atopic dermatitis (4), asthma (5), and arthritis (6) among others. Although MCs are classically activated by the antigen bound immunoglobulin (Ig)E and high affinity IgE receptor (FcεRI) complex, MCs are also activated by G protein-coupled receptors (GPCR). Of particular interest is the activation of MCs through the mas related G protein-coupled receptor X2 (MRGPRX2). Since its recent identification as the receptor responsible for pseudoallergic reactions (7), MRGPRX2’s role in atopic dermatitis, psoriasis (8), asthma (9), and drug allergies (10) has been explored. Predominantly expressed by connective tissue MCs (MC_TC_, mast cell that store both tryptase and chymase in their granules) (11), MRGPRX2 is an important biomarker of MCs that is activated by basic secretogogues such as substance P (SP), a neuropeptide involved in allergic inflammation (1, 12).

Since MRGPRX2 is very promiscuous and binds several different protein ligands and small molecules like proadrenomedullin N-terminal peptide, PAMP-12, albumin fragments, and SP, among others (13), it is unclear precisely where these ligands bind the MRGPRX2, and how they interact at a molecular level. Recent break through cryogenic electron microscopic (cryoEM) analyses have described the structure of MRGPRX2 receptor in complex with its ligands like compound 48/80, cortistatin-14, PAMP-12, and SP, and highlight the two distinct sub pockets within the ligand-binding domain of the receptor (14, 15). A highly electronegative sub pocket comprised of Asp184 and Glu164, and a hydrophobic pocket consisting of Trp243 and Phe170, characterize the binding pocket of the receptor. It was shown that mutations at these positions either inhibited or significantly reduced the activity of MRGPRX2 against its agonist compound 48/80 (14). In parallel, the complementary structures of MRGPRX2 ligands have also been examined. It has been shown that the presence of basic and hydrophobic residues within a peptide ligand (PAMP-12 and cortistatin-14), and in a specific peptide sequence, is required for MRGPRX2 activation (16, 17). Although these studies show the importance of amino acid composition on ligand interactions with MRGPRX2, the interaction of SP with MRGPRX2 and its association with specific functional outcomes, such as degranulation and cytokine release is still poorly understood.

In the present article we have examined the activation of MRGPRX2 on human MCs by SP and its analogs. SP is a neuropeptide that regulates several inflammatory diseases such as arthritis, inflammatory bowel disease, and asthma. Our results show that SP triggers a concentration dependent activation of the human mast cell line, LAD2. MRGPRX2 activation causes MRGPRX2 internalization, whereby more than 38% of the receptors are internalized after 1 min of activation. We further conducted molecular dynamic (MD) simulations to identify the important intermolecular forces and the important amino acid residues within SP and MRGPRX2 that facilitate binding. Arg1 and Lys3 in SP were deemed important for SP interaction with MRGPRX2, and the hydrogen bonds (h-bonds) and salt bridges governed the interactions. The computational findings were experimentally validated using SP analogs, wherein, the peptide sequences devoid of Lys3 failed to activate LAD2 cells, while mutations at other sites in SP had no effect on peptide activity in activating mast cells. Furthermore, we show that modulation in the amino acid residues within SP can help in designing potential antagonists of SP, which hold immense potential in therapeutics.

## Materials and methods

### Cell culture

The LAD2 cell line (a gift from Arnold Kirshenbaum and Dean Metcalfe from the National Institutes of Health, NIAID) was cultured in StemPro-34 SFM media (Life Technologies, Burlington, ON, Canada) supplemented with 2mM L-glutamine, 100 U/ml penicillin, 50 mg/ml streptomycin, and 100 ng/ml recombinant human SCF (Peprotech, Rocky Hill, NJ, USA). Cells were maintained at 1×10^5^ cells/ml at 37°C and 5% CO_2_ and periodically tested for the expression of CD117 (eBioscience, Invitrogen Carlsbad, CA, USA), and FcϵRI (eBioscience, Invitrogen Carlsbad, CA, USA) by flow cytometry using a CytoFlex flow cytometer (Beckman Coulter, Brea, CA, USA).

### Degranulation analysis

Degranulation was determined by measuring the release of the granule-associated β-hexosaminidase (β-hex). Briefly, LAD2 cells were washed with 0.4% BSA-HEPES buffer (10 mM HEPES, 137 mM NaCl, 2.7 mM KCl, 5.6 mM glucose, 5.6 mM Na_2_HPO_4_, 1.8 mM CaCl_2_, and 1.3 mM MgSO_4_ at a pH of 7.4) and 5 × 10^4^ cells were added to each well of a 96 well plate, activated with different concentrations (0.1 to 10 μM) of SP and SP analogs (SP1, SP2, SP3, SP4 or SP5, RS synthesis, Louisville, KY, USA) for 30 min at 37°C and 5% CO_2_; SP (5 μM, Sigma-Aldrich Canada, Oakville, ON, Canada) was included as positive control. For SP analog analysis, sets of cells were preincubated with SP analogs for 30 min, 1h, and 3h at 37°C, 5% CO_2_, followed by 30 min SP treatment. For IgE-dependent activation assays, LAD2 cells were suspended in StemPro-34 SFM media at a cell density of 5 × 10^4^, and sensitized with 0.5 μg/mL biotinylated IgE overnight at 37°C and 5% CO_2_, and challenged with 0.1 μg/mL streptavidin for 30 min at 37°C and 5% CO_2_. Untreated LAD2 cells were included as negative controls. After activation, LAD2 cells were centrifuged at 200 × *g* for 5 min, and cell-free supernatants were collected in a different 96 well plate; cell fractions were resuspended and lysed with 0.1% Triton X-100. β-hex release was measured by the hydrolysis of p-nitrophenyl-N-acetyl-β-D-glucosamine (Sigma-Aldrich Canada, Oakville, ON, Canada) in 0.1 M sodium citrate Buffer (pH 4.5), and analyzed using a Biotek Synergy H1 microplate reader (Agilent Technologies, Inc. Santa Clara, CA, USA). Results are reported as the percentage of intracellular β–hex released into the 0.4% BSA-HEPES buffer after correction for spontaneous release.

### Histamine release

Histamine release was measured using *o*-phthalaldehyde as an indicator. Briefly, 1 × 10^5^ LAD2 cells were pelleted at 200 × *g* for 5 min, washed with HEPES buffer, and resuspended in 100 μL HEPES buffer in presence of the indicated concentrations of SP analogs, and incubated for 30 min at 37°C and 5% CO_2_. SP, and untreated cells were included as positive and negative controls respectively. Cells were pelleted at 200 × *g* for 5 min, and 60 μL of supernatant were transferred into a black 96 wells microplate (Greiner, Swedesboro, NJ, USA), 12 μL of 1M NaOH were added to the wells, followed by 2 μL of *o*-phthaldialdehyde dissolved in methanol (Sigma-Aldrich Canada, Oakville, ON, Canada) and incubated for 4 min at RT. The reaction was stopped by the addition of 6 μL of 3M HCl. Fluorescence intensity was measured using a 360 nm excitation, and 450 nm emission filters in a BioTek Synergy H1 microplate reader (Agilent Technologies, Inc. Santa Clara, CA, USA). Histamine released was measured by the interpolation from a histamine dihydrochloride (Sigma-Aldrich Canada, Oakville, ON, Canada) standard (8- 500 ng/mL in HEPES buffer) curve. Lower limit of detection for this assay is approximately 5-7 ng/ml (18). For SP analog studies, cells were preincubated with the indicated SP analogs for as indicated, and then were activated by SP for 30 min for the analysis of histamine.

### Chemokine and cytokine (CCL2 and TNF) release

LAD2 cells (2 × 10^6^/mL) were incubated for 24 h with 0.1, 1, and 10 μM concentrations of SP and SP analogs, at 37°C and 5% CO_2_, and cytokine levels of TNF and CCL2 released in the cell-free supernatants were quantified using commercial enzyme-linked immunosorbent assay (ELISA) according to the manufacturer’s (ThermoFisher Scientific, Waltham, MA, USA) instructions. SP-activated cells (5 μM), and untreated cells were included as positive and negative controls respectively. For SP analog studies, cells were preincubated as indicated, and then were activated by SP for 30 min and 24 h for the analysis of CCl2 and TNF.

### Analysis of MRGPRX2 expression by flow cytometry

For the analysis of MRGPRX2 expression, LAD2 cell suspensions were seeded at a density of 2 × 10^5^ cells/mL, and incubated with 5 µM of SP for 1 to 60 min at 37°C and 5% CO_2_. Cells were washed twice with 0.1% BSA-PBS buffer at 200 × *g* for 5 min, resuspended in 0.1% BSA-PBS buffer, and incubated for 1 h in the dark at 4°C with anti-human MrgprX2-PE (Biolegend, San Diego, CA, USA); or anti-human FcϵRIα-APC (eBioscience, Invitrogen Carlsbad, CA, USA); washed twice with 0.1% BSA-PBS buffer at 200 × *g* for 5 min, and fixed with 5% formalin neutral buffered solution (Sigma-Aldrich, Oakville, ON, CAN), for 15 min at RT followed by the addition of 3% BSA-PBS buffer, mixed and centrifuged at 200 × *g* for 10 min at 4°C, cells were then resuspended in 0.1% BSA-PBS buffer, and analyzed on a CytoFlex flow cytometer (Beckman Coulter, Brea, CA, USA). Mouse IgG2b, k isotype control-PE, and Armenian hamster IgG isotype control-APC (eBioscience, Invitrogen Carlsbad, CA, USA) were included as negative controls. Expression of MRGPRX2, and FcεRIα were analyzed using FlowJo v.10.8 software (BD Life Sciences, Ashland, OR, USA) and compared to control values. Results are reported as histograms and mean fluorescent intensity (MFI ± SEM).

### SP and MRGPRX2 structure generation

The initial conformation of MRGPRX2 used in our molecular dynamics simulations was taken from K. Lansu et al, 2017 (19). The truncated structural model of MRGPRX2 in complex with dextromethorphan or ZINC-9232 was used in our study. SP was docked into the MRGPRX2 by the SMINA fork of AutoDock Vina software (20, 21). Nine different complexes were obtained and examined. MRGPRX2 was inserted into Dioleoylphosphatidylcholine (DOPC) membrane for further simulations. The starting conformation of the DOPC membrane was prepared with CHARRM-GUI (http://www.charmm-gui.org) (22). The prebuilt membrane was then equilibrated in TIP3P (computational water model) water with NPT (constant-temperature, constant-pressure ensemble) molecular dynamics (MD) simulation for several nanoseconds using GROMACS simulation package supplied with recently reported OPLS-aa parameters for lipids (23, 24). MRGPRX2 was inserted into the DOPC membranes using a inflation methodology (25).

### Molecular docking calculations

Preferred binding modes of the SP to the aforementioned structure of MRGPRX2 was determined by molecular docking with SMINA version of the AutoDock Vina software. AutoDock Tools was used to prepare the input files (PDBQT) for SP and MRGPRX2. MRGPRX2 was kept rigid while the ligands were flexible. The dimensions of the grid box in the docking software setup were set to 100 × 100 × 100 points with a spacing of 0.374 Å. The center of the box was set to the same as that used for small molecules docked in MRGPX2 by K. Lansu et al, 2017. For enhanced accuracy and reproducibility of the predictions, the docking was performed with 1024 exhaustiveness parameter. The scoring function of the AutoDock Vina described was used to obtain the docking modes of SP.

### Molecular dynamic simulations

The MD simulations of the SP-MRGPRX2 complexes were performed using GROMACS 2018 software (23). The OPLS-aa force field was selected for the peptide-protein complexes (26, 27). Amino acids were parametrized using the GROMACS pdb2gmx program. The starting structure for each SP-MRGPRX2 complex was solvated in a periodic box of size 70.4 × 70.1 × 171.5 Å. The box was elongated along the z-axis to allow space for pulling SP out of the receptor. The water molecules inserted into the lipid bilayer were manually deleted. The TIP3P water model was applied to water molecules (28). Periodic boundary conditions were applied in all three directions with the explicit solvent model. Chloride ions were included to neutralize the system. The final number of atoms in the simulations including the peptide and water was about 70,000. The Maxwell distribution was employed to determine the initial velocities at 310 K. Equilibration consisted of short NVT (constant-temperature, constant-volume ensemble) and NPT simulations for 0.1 and 1 nanoseconds (ns), respectively. The V-rescale thermostat was used to keep the temperature constant by coupling to a reference temperature of 310 K during the NVT simulations (29). Nose-Hoover thermostat with semi-isotropic Parrinello-Rahman pressure coupling to a pressure of 1 bar was used during the NPT simulations (30). Coupling times of 0.5 and 2.0 picosecond (ps) were used for the thermostat and barostat, respectively. The isothermal compressibilities were set to 4.5 × 10^-5^ bar^-1^ in both x-y and z-directions of the semi-isotropic barostat.

Electrostatic interactions were determined using the Particle Mesh Ewald (PME) method (31). A cutoff of 12 Å was applied for van der Waals interactions. 5000 steps of energy minimization were carried out with the steepest descent algorithm before the equilibration. The two stages of equilibration (NVT and NPT) were performed without any restraints on the coordinates of the peptide and protein. Next, 30 ns NPT simulations were performed for all structures. Every 2 ps, the atomic coordinates were saved for subsequent analysis. Additionally, the stability of the docked SP-MRGPRX2 complexes were checked in separate MD simulations. Two stages of equilibration (NVT and NPT) were performed with a spring-like positional restraints (force constant of 5000 kJ mol^-1^ nm^-2^) applied on the coordinates of the backbone C-atoms of SP and MRGPRX2 during the NVT equilibration. The final NPT simulations were performed for 1 ns without any constraints. Root mean square distance (RMSD) between the initial and the final SP configurations were compared to assess the stability of the binding poses predicted by the docking calculations.

### Free energy calculations

Binding free energies of the docked SP-MRGPRX2 complexes were estimated with umbrella sampling methodology (31). Special MD simulations were performed in which the peptide was gradually pulled away from the complex to a distance of approximately 5 nm during a time of 1 ns (force constant of 2000 kJ mol^-1^ nm^-2^). Next, 1000 different configurations of the system at different distances between SP and MRGPRX2 were extracted from the trajectory, and a set of 200 configurations was selected as initial configurations for umbrella sampling simulations. Umbrella sampling free energy calculations consisted of performing around 200 simulations at constrained (fixed) distances between the ligand and the receptor. The harmonic constraint force was applied to the center of mass of the ligand with constant of 2000 kJ mol^-1^ nm^-2^. First, the 200 systems were equilibrated in NPT ensemble for 1 ns. Next, the actual production simulations were performed for 2.5 ns during which the force acting on the ligand was sampled. The sampled forces were then used in the weighted histogram analysis using *wham* software supplied in the GROMACS package to produce the free energy profiles (32). For every studied complex, the distance histograms between the protein and the ligand were manually checked to ensure proper overlap of the histograms and resulting accuracy of the results. The differences in the minimal and the maximal values of the profiles are then used as estimates of the binding free energies of the complexes. It should be noted that the actual sampling of the forces that are used for the free energy calculations is performed in fully equilibrated separate umbrella sampling runs, which might be regarded as a limiting case of infinitely slow pulling (33).

### Statistical analysis

All cell activation experiments were performed in duplicate and represent at least three independent biological replicates (n ≥  3). Values are expressed as mean ± SEM. All statistical analyses were performed using GraphPad Prism statistical software (GraphPad, Sand Diego, CA, USA). Statistical differences in the mean values among treatment groups were determined by using a one-way analysis of variance (ANOVA) test with *post hoc* analysis with Tukey’s multiple comparison tests. In all cases, a value for *P* < 0.05 was considered statistically significant.

## Results

### SP trigged mast cell activation is accompanied by MRGPRX2 internalization

**Figure 1:**
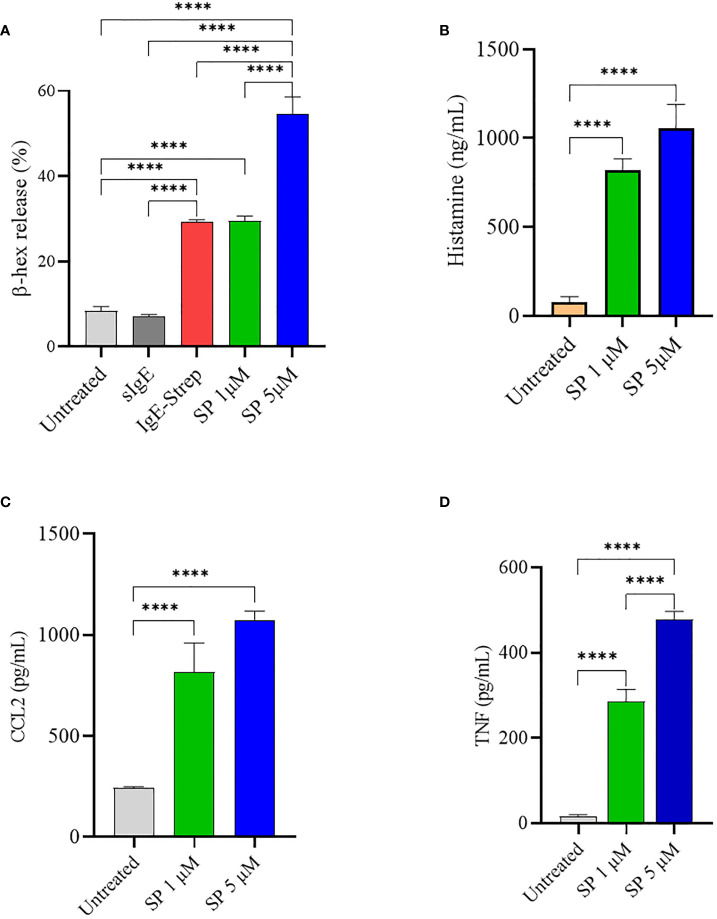
SP stimulates LAD2 degranulation and release of proinflammatory mediators. LAD2 cells were activated with SP for 30 min (1 μM and 5 μM). For comparison, LAD2 were sensitized with IgE (0.5 μg/mL) overnight and challenged with streptavidin (0.1 μg/mL) for 30 min. β-hex release **(A)** and histamine release **(B)** were measured as described in Methods. CCL2 **(C)** and TNF **(D)** production after SP activation for 24 h was measured by ELISA. Data is presented as mean ± SEM (n = 4, ****p < 0.0001).

SP has been shown to induce mast cell activation and degranulation through MRGPRX2 (34). LAD2 cells were activated with increasing concentrations of SP and degranulation was quantified by measuring β-hex release. Untreated cells and IgE sensitized cells did not degranulate and displayed only background levels of β-hex release of approximately 7-9% of total β-hex stored in the granules. FcεRI activated LAD2 (activated with biotinylated IgE and streptavidin) showed approximately 29% degranulation. As expected, SP caused a concentration-dependent release of β-hex (**Figure 1A**) and stimulation with 5 µM SP resulted in 55% release of β-hex. MCs contain several different classes of granules which contain different mediators (2, 3). Depending upon the stimulus, MCs release different sets of granules in a process known as piecemeal degranulation. Histamine is stored in distinct granules and is sometimes released independently of β-hex (3). We therefore evaluated histamine released upon SP triggered LAD2 degranulation (**Figure 1B**). SP (1 and 5 µM) activated significant histamine release compared to the untreated control. SP also activated the production of CCL2 and TNF by LAD2 (**Figures 1C, D**, respectively).

**Figure 2:**
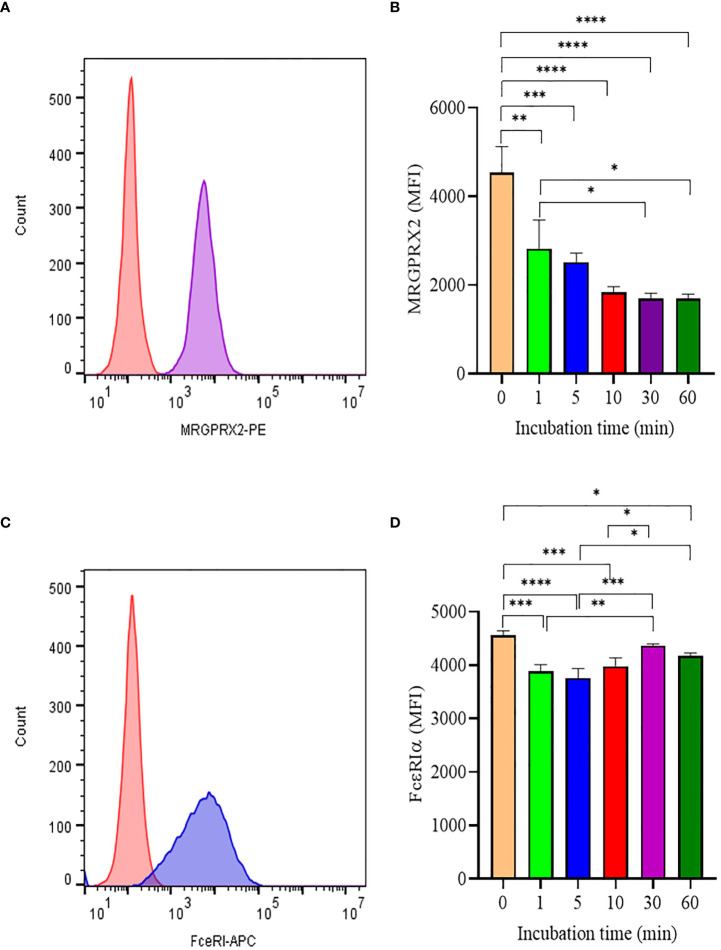
Analyis of MRGPRX2 and FcεRIα surface expression by flow cytometry. **(A)** LAD2 cells were treated with 5 μM SP and MRGPRX2 expression was assessed after 60 min (MRGPRX2 is purple curve; isotype control is red curve). **(B)** MRGPRX2 expression after 1, 5, 10, 30 and 60 min activation with SP as measured by mean fluorescence intensity (MFI). **(C)** Expression of FcεRIα after activation with SP as in **(B)** (FcεRIα is the blue curve; isotype control is the red histogram). **(D)** FcεRIα expression after 1, 5, 10, 30 and 60 min activation with SP as measured by MFI. Data is presented as mean ± SEM (n = 3, *p < 0.01, **<p 0.05, ***p < 0.001, ****p < 0.0001).

Like most GPCR, MRGPRX2 is internalized upon activation and initiates signaling through the β-arrestin pathway (12). To better understand how SP triggered MRGPRX2 internalization, we analyzed surface MRGPRX2 expression following SP activation using flow cytometry. When activated with SP, surface expression of MRGPX2 decreased rapidly (within 1 min), reaching minimal levels by 10-30 min (**Figures 2A, B**). In contrast, the treatment of LAD 2 with 5 µM SP showed no major changes in FcεRI expression with time (**Figures 2C, D**). The results indicate that the effect of SP is specific to MRGPRX2

### SP-MRGPRX2 interaction is governed by h-bonds and salt bridges

To better understand mast cell activation through MRGPRX2, it is important to understand the intermolecular forces governing the interactions. Therefore, we examined the conformational and energetic relationships between SP and MRGPRX2. The probable poses of SP in the binding pocket of MRGPRX2 were determined (**Supplementary Figure 1**) and were refined with MD simulations and free energy integrations.

### Stability and structural analysis

**Table 1** lists the binding affinity and the RMSD (before and after simulation) of the various poses of SP in the binding pocket of MRGPRX2 after 1ns MD simulation. Though all of the SP poses in the SP-MRGPRX2 complexes were stable (binding affinity of -9.5 - -9.1 for Complexes 1 – 9, **Table 1**), the SP-MRGPRX2 Complex 1 was found to be most stable in terms of most negative binding affinity (-9.5 kcal/mol) and showed lowest deviation from the initial position of the corresponding SP conformation (RMSD = 0.143 nm). To further substantiate the stability of the complexes, the distance between the center of masses of the corresponding SP pose and the MRGPRX2 before and after the short MD simulations for each SP-MRGPRX2 complex was determined (**Table 1**). Closer position of the ligand to the receptor suggests stronger interaction and hence a larger binding affinity. As seen in **Table 1**, the SP pose in Complex 1, which had the most negative binding affinity, was the closest to MRGPRX2 after equilibrium. The distance between the two centers of masses in Complex 1 was found to be 1.39 nm after simulation, causing a change of 0.015 nm from the initial SP position. Interestingly, the position of SP in Complex 9 was found to be stable as well. The center of mass of SP in Complex 9 changed only by 0.017 nm after equilibrium. These results suggest that SP poses in Complex 1 and Complex 9 are stable with a high negative binding affinity and stable position in the SP-MRGPRX2 complex.

**Table 1:** Binding parameters determined computationally for various SP-MRGPRX2 complexes in the column order of i) SP-MRGPRX2 complexes, ii) values of the binding affinities of the docked SP-MRGPRX2 complexes predicted by SMINA using AutoDock Vina scoring functions, iii) root mean square deviation (RMSD) between the initial and the final states of the ligand after 1 ns of MD simulations, iv – vi) distances, D between the centers of mass of the ligand and the receptor of the SP-MRGPRX2 complexes at the initial (Init. D) and the final (Equil. D) states of the MD simulations, and vii) free energy differences (F.E. Diff) between the docked and undocked (water-solvated) states of the peptide as predicted by the umbrella sampling simulations for the SP-MRGPRX2 complexes.

A.D. Vina	Bind. Affinity, kcal/mol	RMSD, nm	Init. D., nm	Equil. D., nm	Δ, nm	F.E. Diff., kcal/mol
Complex 1	-9.5	0.143	1.376	1.391	0.015	59.5
Complex 2	-9.4	0.235	1.726	1.864	0.138	43.3
Complex 3	-9.3	0.146	1.691	1.724	0.033	42.4
Complex 4	-9.3	0.22	1.756	1.861	0.105	37.1
Complex 5	-9.2	0.22	1.742	1.867	0.125	34.6
Complex 6	-9.2	0.163	1.52	1.563	0.043	59.2
Complex 7	-9.2	0.295	1.799	1.894	0.095	56.2
Complex 8	-9.2	0.164	1.758	1.803	0.045	48.5
Complex 9	-9.1	0.197	1.508	1.525	0.017	91.4

RMSD= root mean square deviation.

Next, the relative binding free energy of the various SP poses were determined by mechanically pulling the corresponding SP pose from the SP-MRGPRX2 complexes. Binding free energy for every complex was estimated as the difference between the maximal and the minimal free energy values. Separate umbrella sampling calculation was performed for every SP-MRGPRX2 complex after 30 ns of NPT MD simulation. The resulting free energy profiles of the umbrella sampling are presented in **Supplementary Figure 2**, and the corresponding binding free energies are presented in **Table 1**. In accordance with the previous results, Complex 9 showed the highest binding free energy of 91.4 kcal/mol, and was followed by Complex 1 (59.5 kcal/mol). These results suggest that SP poses in Complex 1 and Complex 9 are closest to the MRGPRX2 receptor, were tightly bound, and were positioned deep in the binding pocket of the receptor. Deeper positioning of the complex in the binding pocket of the receptor allows for more interactions between the ligand and the receptor and, consequently, for higher binding free energies.

### H-bonds and salt bridges between the peptide and the protein in each complex

Careful analysis of possible h-bonding and other intermolecular forces such as salt bridges are required for more insights in to SP-MRGPRX2 interactions. We examined the total number and the total active time of the h-bonds formed in each of the simulated SP-MRGPRX2 complexes. The h-bonds subprogram of GROMACS package was run on the 30 ns simulation trajectory of the membrane-inserted SP-MRGPRX2 complex. The total number of h-bonds for each complex active for 10 and 50% of simulation time are listed in **Table 2**. As expected, SP in Complex 1 formed the highest numbers of highly active h-bonds. Nineteen h-bonds in the SP-MRGPRX2 Complex 1 were active for more than 10% of simulation time, while 9 h-bonds were active for more than 50% of the simulation time. Complex 1 was followed successively by Complex 2 with 16 h-bonds active for more than 10% of simulation time and 8 h-bonds active for than 50% of simulation time. Complex 9 formed 14 h-bonds that were active for more than 10% of simulation time and 8 h-bonds that were active for more than 50% of simulation time.

**Table 2:** Intermolecular forces governing the interaction of SP and MRGPRX2 receptor, in the column order of i) SP-MRGPRX2 complexes, ii) numbers of active h-bonds for more than 10% (N(THB)>10%) of simulation time, and iii) the number of highly active h-bonds for more than 50% (N(THB)>50%) of the total simulation time, iv-v) salt bridges (attractive force between two oppositely charges molecules) formed between the SP and MRGPRX2 detected by the VMD software package, and the corresponding average distances between the residues participating in the salt bridges for every studied complex.

A.D. Vina	*N* (*T_HB_* >10%)	*N* (*T_HB_* >50%)	Salt Bridges	Average Distance, nm
Complex 1	19	9	GLU164-ARG1	4.501
Complex 2	16	8	ASP174-ARG1	4.526
Complex 3	9	1		
Complex 4	19	2		
Complex 5	7	4	ASP174-LYS3 ASP247-ARG1	5.67 9.474
Complex 6	9	4		
Complex 7	14	3	ASP174-LYS3	8.179
Complex 8	16	2	GLU29-LYS3 ASP174-ARG1	5.411 4.258
Complex 9	14	7	GLU164-LYS3 ASP184-ARG1	2.74 4.565

Furthermore, we examined the trajectories of the MD simulations for possible salt bridges between SP and MRGPRX2 in the given SP-MRGPRX2 complexes. The salt bridges were automatically determined by an extension supplied with the Visual Molecular Dynamics (VMD) software package (35), which also measures the distance between the charged residues forming the salt bridge for every frame of the MD simulation trajectory. The detected salt bridge and the average distances between the charged amino acid residues for every complex are presented in **Table 2**. As can be seen from the table, the studied complexes formed only 1-2 salt bridges between SP and MRGPRX2 during the simulation time. The average distance between the participating residues were larger than 4 nm except Complex 9, wherein the average distance between Lys3 of SP and Glu164 of MRGPRX2 that formed salt bridge was 2.74 nm. These results indicate that the salt bridges (electrostatic interactions) were largely inactive, and the resulting interaction was rather weak (36). Complex 9 was the only SP-MRGPRX2 complex which formed stable salt bridge with the average distance between the residues less than 4 nm (36). Thus, in addition to a robust number of h-bonds, stable salt bridges modulate the strong interaction of the SP with MRGPRX2 receptor in Complex 9. We further identified the important amino acid residues in SP and MRGPRX2 in Complex 9 that participated in the formation of h-bonds (**Table 3**). Interestingly, the corresponding residues within SP and MRGPRX2, which formed salt bridges in Complex 9, also participated in h-bond interactions. **Table 3**, row 10 and 11 show that SP Lys3 and MRGPRX2 Glu164 formed active h-bonds for 67% and 31% of the simulation time respectively. Similarly, SP Arg1 and MRGPRX2 Asp184 formed active h-bonds. Gln5 and Gln6 of SP showed h-bond interactions with Phe170 and Asn85 of MRGPRX2 respectively. Thus, due to the stable salt bridge and the high number of h-bonds, the configuration of the peptide in Complex 9 seems to be the most probable among the ones studied in this contribution.

**Table 3:** H-bonds between SP and MRGPRX2 in Complex 9 together with the percent of time they were active during the simulation obtained with gmx hbonds software.

	Donor	Hydrogen	Acceptor	% of Time Active
1	ND2@ASN85	HD21@ASN85	OE1@GLN6	88%
2	OH@TYR89	HH@TYR89	O@GLN6	31%
3	OG@SER257	HG@SER267	O@GLN5	10%
4	N@ARG1	H1@ARG1	OD1@ASP184	88.40%
5	N@ARG1	H1@ARG1	OD2@ASP184	12.60%
6	N@ARG1	H1@ARG1	OG1@THR187	87.50%
7	NE@ARG1	HE@ARG1	O@LEU163	29.60%
8	NH1@ARG1	HH11@ARG1	OD1@ASP184	10.80%
9	NH1@ARG1	HH11@ARG1	OD2@ASP184	89.50%
10	NZ@LYS3	HZ1@LYS3	OE1@GLU164	67.40%
11	NZ@LYS3	HZ1@LYS3	OE2@GLU164	31.30%
12	NZ@LYS3	HZ1@LYS3	O@CYS168	26.60%
13	NE2@GLN5	HE21@GLN5	O@PHE170	66.90%
14	NE2@GLN6	HE21@GLN6	OG@SER257	57.40%

### Ligand binding/unbinding reaction pathway

A steered MD simulation was performed by slowly pulling the SP out of the receptor for 5 nm during a time period of 1 ns. The analysis of the pulling process provides a rough model of the binding/unbinding of the peptide to/from the receptor. We examined the changes in the number of h-bonds and salt bridges between the SP and MRGPRX2 receptor during the binding/unbinding process. **Supplementary Figure 3** presents the number of h-bonds between SP and MRGPRX2 dependent on the distances between the centers of mass of SP and MRGPRX2 sampled during the pulling simulations. It could be seen that, while for the majority of the complexes the number of h-bonds gradually decays to zero shortly after the distance of 4 nm, Complex 1 and 9 depict a nonzero number of h-bonds even at a distance of 5 nm. This observation clearly suggests that some flexible part of the protein extends and follows the peptide during the unbinding (pulling) process.

**Figure 3:**
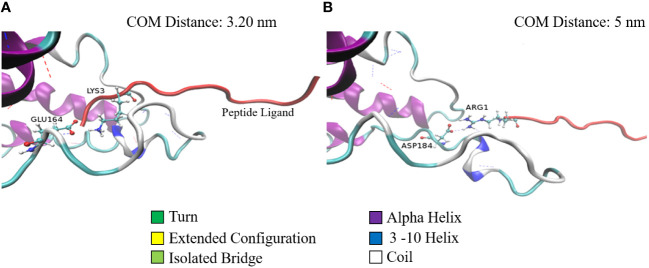
Two configurations of Complex 9 captured during the pulling simulation. Configurations of Complex 9 correspond to the center of mass distance of 3.2 nm **(A)** and 5.0 nm (**B**). MRGPRX2 (colored according to its secondary structure) and SP (red colored) are represented by cartoons (special VMD drawing method). Salt-bridge residues are represented by ball and stick models. Dashed lines denote h-bonds between the proteins.

A similar phenomenon is observed with respect to the salt bridges as well (**Figure 3**). **Figures 3A, B** show the stable salt bridges formed at a distance of 3.2 and 5 nm between centers of masses of SP and MRGPRX2 in Complex 9. Complex 9 is characterized by two salt bridges between residues Glu164 and Asp184 of MRGPRX2 and Lys3 and Arg1 of SP, respectively. The lifetime of the first and highly stable Glu164-Lys3 salt bridge extends from the initial configuration to a distance of about 3.7 nm. Salt bridge Asp184-Arg1 in Complex 9 appears to be initially inactive (residue distance more than 0.4 nm) but is activated for a significant range (~2 nm) of distances with values between approximately 4.25 and 5.25 nm.

### SP analogs devoid of key residues fail to activate LAD2 cells

**Figure 4:**
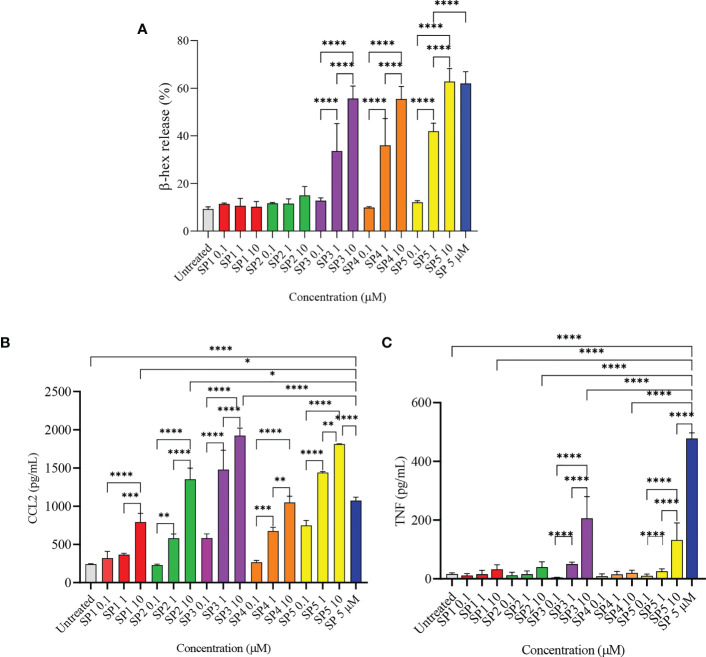
Release of immune mediators by LAD2 cells stimulated with SP analogs. **(A)** LAD2 cells were activated with SP analogs (SP1, to SP5) for 30 min, and β-hex release was measured. **(B)** CCL2, and **(C)** TNF release were analyzed after activation of LAD2 with SP analogs or SP (5 μM) for 24 h Untreated cells were included as negative controls. Data is presented as mean ± SEM (n = 5 for β-hex release, n = 3 for CCL2 release, and n = 4 for TNF release, *p<0.01, ** <p0.05, ***p<0.001, ****p<0.0001).

To confirm the results of MD simulation, and in accordance with other published data, a series of SP analogs (**Supplementary Table 1**) were tested for their activity against LAD2 cells. **Figure 4A** shows the degranulation induced by SP analogs. The results show that replacing Lys3 and Gly5 from SP (SP1 and SP2) greatly reduces the activity of the peptides. The degranulation of LAD2 upon stimulation by 0.1, 1, and 10 µM of SP1 and SP2 remained at basal level in comparison to 5 µM SP (62% degranulation). Further, since hydrophobic residues play an important role in ligand-MRGPRX2 interactions (17), Phe8 was replaced with 4-Benzoyl-Phe8 (SP4), as well; Phe7 and Phe8 were simultaneously replaced with 4-Chloro-Phe7 and 4-Choloro-Phe8 (SP5), respectively. Results showed that altering the hydrophobicity of amino acids did not alter the activation potential of the peptides. The degranulation of LAD2 upon stimulation by 0.1, 1, and 10 µM of SP4 were found to be 10, 36, and 55%, respectively, against 62% degranulation by 5 µM SP. Similarly, the degranulation of LAD2 upon stimulation by 0.1, 1, and 10 µM of SP5 were found to be 12, 42, and 63%, respectively. SP analog where Gly9 was replaced with Pro9 (SP3), showed no change in activity when compared to SP.

We also measured the production of CCL2 by LAD2 that were activated with the SP analogs (**Figure 4B**). All of the SP analogs elicited a concentration-dependent activation of CCL2 production. However, in contrast to β-hex release, the amount of CCL2 produced by cells activated with 10 µM of the peptides was either comparable or higher than that released by SP (5 µM). CCL2 released by 10 µM SP1 - 5 were 792, 1352, 1924, 1050, and 1813 pg/mL, respectively, in comparison to 1074 pg/mL CCL2 released by cells activated with 5 µM SP. This is in contrast to the negligible β-hex release by SP1 and SP2 when compared to SP. Furthermore, and surprisingly, only SP3 and SP5 (10 µM) triggered the release of TNF from the activated cells (**Figure 4C**). These results suggest that the level of degranulation (β-hex) and cytokine release is not directly comparable when cells are activated through MRGPRX2 by these peptides.

**Figure 5:**
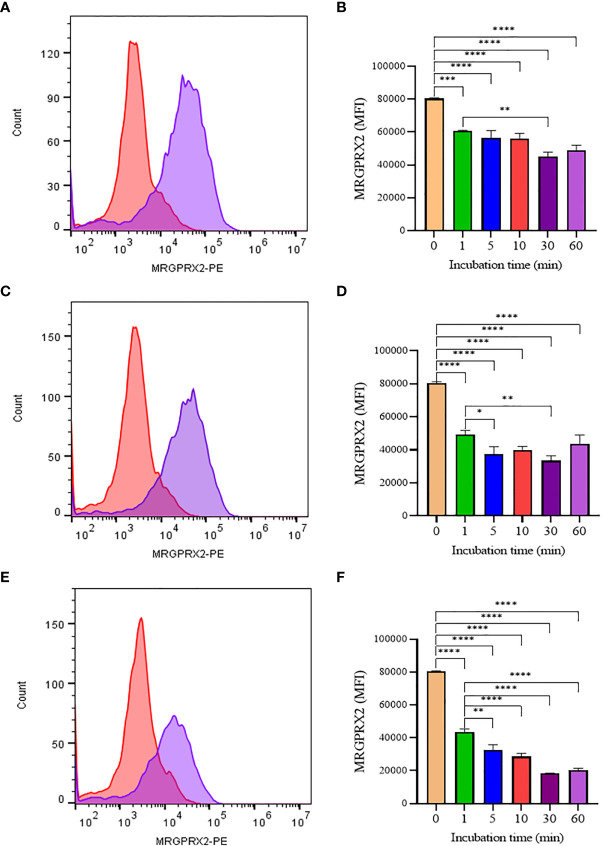
Flow cytometry analysis of SP analogs on MRGPRX2 expression in LAD2 cells. Histograms showing MRGPRX2 internalization effect after 60 **min** SP analogs treatment (A, SP1; C, SP2; E, SP4). LAD2 cells were treated overtime (1 to 60 min) with SP analogs (B, SP1; D, SP2; F, SP4), and MRGPRX2 expression was compared to untreated cells. Data is presented as mean ± SEM (n = 3, *<0.05, **<0.01, ***<0.001, ****<0.0001).

**Figure 6:**
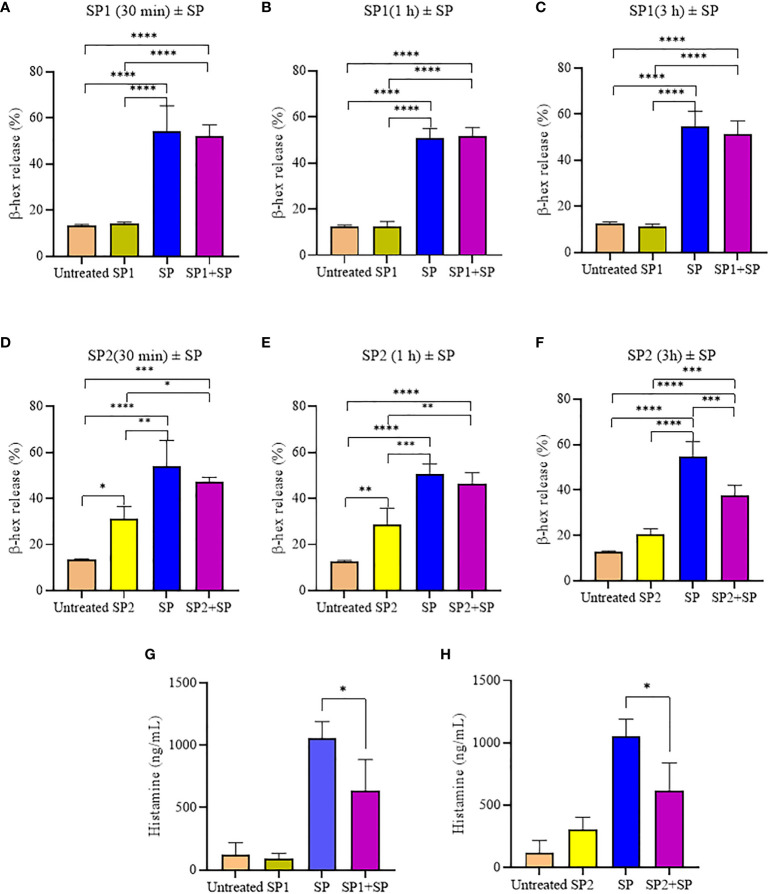
Inhibitory effect of SP analogs on the degranulation of LAD2 cells by the 30 **min** activation by SP. **(A-C)** Time dependent effect of SP1 preincubation on the β-hex release from the SP activated LAD2 cells. **(D-F)** Time dependent effect of SP2 preincubation on the β-hex release from the SP activated LAD2 cells. **(G-H)** Effect of 180 min SP1 and SP2 preincubation on the histamine release from the SP activated LAD2 cells. Untreated and SP treated β-hex and histamine values are included as controls in all tested conditions. Data is presented as mean ± SEM (n = 4, *p<0.01, **p<0.05, ***p<0.001, ****<0.0001).

To confirm that the activity of SP analogs is mediated through MRGPRX2, we studied the effect of SP analogs (SP1, SP2 and SP4) on the surface expression of MRGPRX2 on LAD2 cells. Our results (**Figure 5**) show that SP derived peptides, like SP, caused an immediate and rapid decrease in the MRGPRX2 expression on the surface of LAD2. The results confirm that the activity of SP analogs is mediated through MRGPRX2. Further, since SP1 and SP2 activated less β-hex release than SP, it is possible that these peptides bound MRGPRX2 less efficiently and therefore could be used as competitive inhibitors of SP. To study this, LAD2 cells were preincubated with 10 µM of SP1 and SP2 for 30, 60 and 180 min and then were activated with 5 µM SP for 30 min (**Figure 6**). β-hex was measured as a gauge of degranulation. Results show that pretreatment of LAD2 with SP2 reduced the β-hex released by SP, and the effect was more pronounced at a longer incubation time (180 min). One hundred eighty min of pre-incubation with SP2 reduced the β-hex released by SP by 33% (**Figure 6F**). Since the SP analogs were more effective at 180 min, we also measured the histamine released by SP upon pre-incubation with SP1 and SP2 (**Figures 6G, H**). Both SP1 and SP2 reduced the histamine released by SP. Where SP1 reduced the histamine released by 40%, SP2 reduced the histamine released by 42%. These results suggest that SP1 and SP2 could be used as a therapeutic to suppress the inflammation caused by SP.

**Figure 7:**
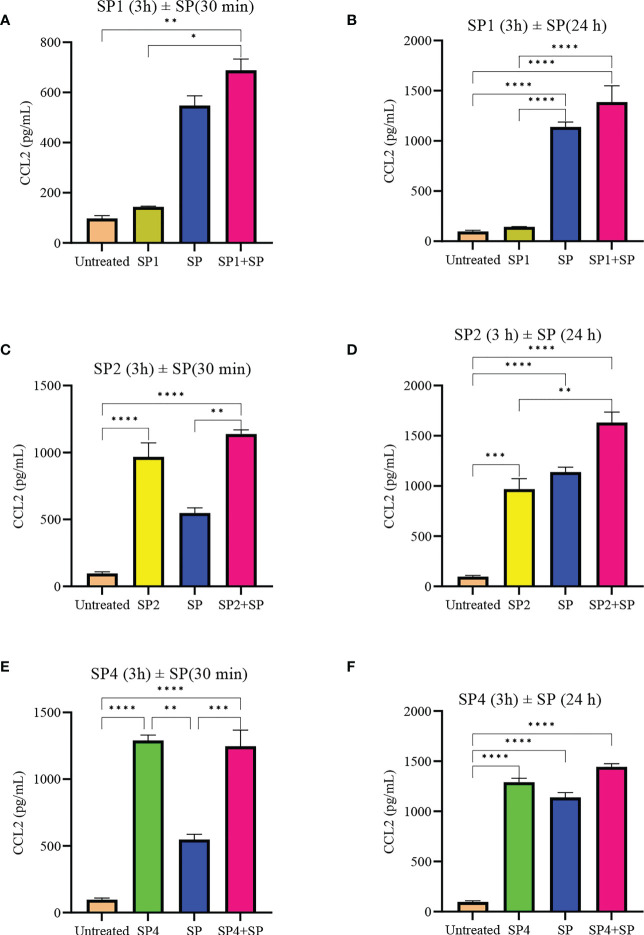
Inhibitory effect of SP analogs on the release of preformed and *de novo* synthesized CCL2 from SP activated LAD2 cells. LAD2 cells were preincubated with SP analogs for 3h and then were activated with SP for the mentioned amount of time. Data showing CCL2 release by the SP analog alone refers to the release of CCL2 due to the preincubation of LAD2 cells with SP analogs, and was measured after the 3h SP analog incubation. **(A)** Release of preformed CCL2 from the SP activated (30 min) LAD2 cells preincubated with SP1. **(C)** Release of preformed CCL2 from the SP activated LAD2 cells preincubated with SP2. **(E)** Release of preformed CCL2 from the SP activated LAD2 cells preincubated with SP4. **(B)** Release of *de novo* synthesized CCL2 from the SP activated LAD2 cells preincubated with SP1. **(D)** Release of *de novo* synthesized CCL2 from the SP activated LAD2 cells preincubated with SP2. **(F)** Release of *de novo* synthesized CCL2 from the SP activated LAD2 cells preincubated with SP4. Untreated and SP treated values are included as controls in all tested conditions. Data is represented as mean ± SEM (n = 3, *p<0.01, **p<0.05, ***p<0.001, ****<0.0001).

**Figure 8:**
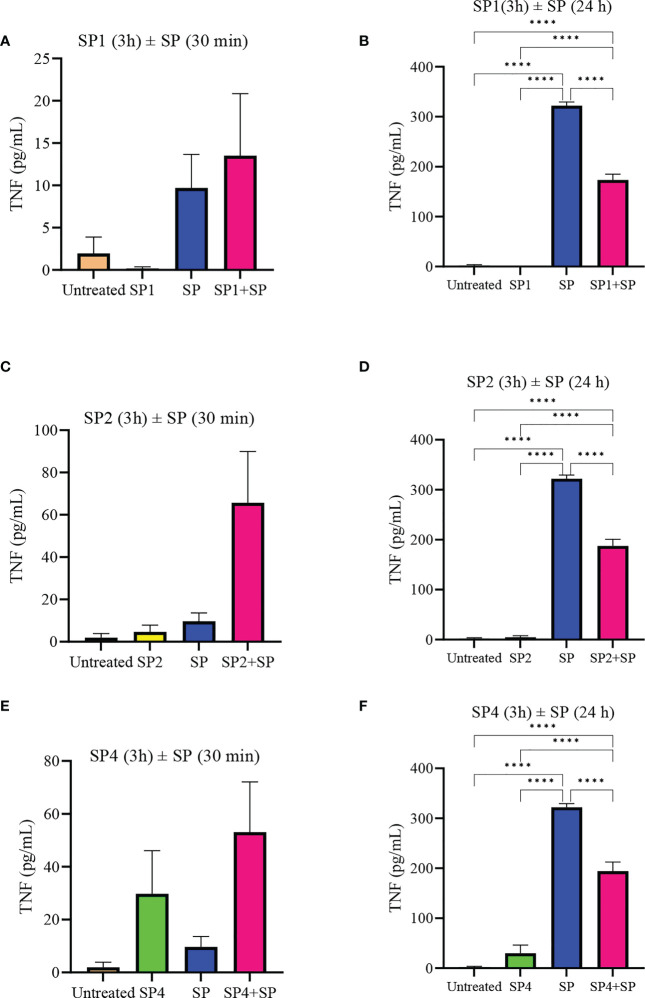
Inhibitory effect of SP analogs on the release of preformed and *de novo* synthesized TNF from SP stimulated LAD2 cells. LAD2 cells were preincubated with SP analogs for 3h and then were activated with SP for the mentioned amount of time. Data showing TNF release by the SP analog alone refers to the release of TNF due to the preincubation of LAD2 cells with SP analogs, and was measured after the 3h SP analog incubation. **(A)** Release of preformed TNF from the SP activated LAD2 cells preincubated with SP1. **(C)** Release of preformed TNF from the SP activated LAD2 cells preincubated with SP2. **(E)** Release of preformed TNF from the SP activated LAD2 cells preincubated with SP4. **(B)** Release of *de novo* synthesized TNF from the SP activated LAD2 cells preincubated with SP1. **(D)** Release of *de novo* synthesized TNF from the SP activated LAD2 cells preincubated with SP2. **(F)** Release of *de novo* synthesized TNF from the SP activated LAD2 cells preincubated with SP4. Untreated and SP treated values are included as controls in all tested conditions. Data is represented as mean ± SEM (n = 3, ****<0.0001).

To further support the result, we also studied effect of SP analog preincubation on the release of CCL2 (**Figure 7**) and TNF (**Figure 8**) from the SP activated LAD2 cells. We included SP4 in our study as well, as it did not trigger TNF release by itself (**Figure 4C**). The effect was studied for the release of both preformed (30 min SP activation, **Figures 7A, C, E** and **8A, C, E**) and *de novo* synthesized cytokines (24 h SP activation, **Figures 7B, D, F** and **8B, D, F**) (37). The results of CCL2 release are in accordance with **Figure 4B**. Preincubation of LAD2 with peptides SP1, SP2 and SP4, triggered the release of CCL2, and successive stimulation with SP added to the total amount of CCL2 for both 30 min and 24 h time periods (**Figure 7**). In contrast, though the preincubation of LAD2 with SP1, SP2 and SP4 had no effect on the release of preformed TNF (**Figures 8A, C, E**), the preincubation of cells with the peptides significantly reduced the *de novo* synthesis of TNF upon SP activation (**Figures 8B, D, F**). The *de novo* synthesis of TNF by SP activated LAD2 preincubated with SP1, SP2 and SP4 was reduced by 46, 42 and 40**%** respectively, with respect to that released by SP alone. The results suggest that manipulation of important amino acid residues in a known ligand of MRGPRX2 receptor could help in developing peptide inhibitors for the receptor which hold immense potential in MRGPRX2 based therapeutics.

## Discussion

The contribution of SP to inflammatory diseases such as atopic dermatitis, asthma, and arthritis has been extensively studied (38). It was presumed that SP activated mast cells exclusively *via* neurokinin-1receptor (NK-1R) but we have shown that the effect of SP can also be mediated through the MRGPRX2 receptor (7). Since each ligand of MRGPRX2 can induce the release of different mediators (39), we studied the important immune modulating molecules that are released from MCs upon SP activation. Our results show that SP activation causes a significant release of histamine, MCP-1/CCL2 and TNF. The results are important as they identify the SP-specific mast cell released mediator that could play an important role in the pathogenesis of inflammatory diseases. The results are in accordance with earlier reported findings, where the activation of LAD2 by SP caused a release of histamine, MCP-1/CCL2 and TNF (40). In the study, the mediators released by SP were compared with those triggered by PAMP-12, and it was reported that PAMP-12 mediated LAD2 activation showed higher release of tryptase. However, the concentrations of SP and PAMP-12 peptides were compared in µg/mL instead of molar concentrations. Since SP and PAMP-12 have different molecular weights, a weight equivalence will result is overall different molar concentrations, and the yielded results could be attributed to the concentration differences. Furthermore, since there was no complete inhibition of one or more mediator, the relative difference in amount of mediator results could also be due to difference in activity of the respective peptide agonists (40).

Receptor internalization is an important event in the downstream signaling pathways, and is initiated when ligands bind MRGPRX2 (12). It has been suggested that ligands with different affinities may also alter the kinetics of internalization, resulting in different signaling and eventually mediators produced (41). Activation with 5 µM SP caused more than 38% of MRGPRX2 to internalize within 1 min of stimulation. The response was time-dependent, but stabilized after 10 min. It has been shown that MRGPRX2 internalization is mediated by the recruitment of β-arrestins. In a study with HEK cells stably expressing β-arrestin2-tobaco etch virus fusion gene and MRGPRX2 receptor, SP induced a concentration dependent recruitment of β-arrestin, and subsequent time-dependent internalization of MRGPRX2 (12). Interestingly, it has been reported that when cells were treated overnight with SP, a subsequent stimulation by SP failed to trigger mast cell activation suggesting that the receptor had been desensitized (12). Furthermore, internalization is mediated *via* parallel endocytosis and micropinocytosis pathways, and upon activation with SP, MRGPRX2 internalizes and is compartmentalized into macropinosomes without degradation (42). These data suggest that it may be possible to desensitize MRGPRX2 using a peptide that induces internalization but does not activate degranulation.

We next used computational simulations to identify the important intermolecular forces that govern the SP-MRGPRX2 interaction, and to simultaneously identify the amino acid residues that are crucial for interaction. H-bonding was the dominant force between SP and MRGPX2 in their interaction. Arg1 and Lys3 were important basic residues within SP that acted as a donor in the h-bond formation, respectively with Asp186 and Glu164 of MRGPRX2 and which were active for 89 and 67% of the simulated time. Apart from these h-bond interactions, the basic residues Arg1 and Lys3 also formed salt bridges with the respective Asp186 and Glu164 and a distance of 4.6 and 2.7 nm between their centers of masses. These interactions were stable and mediated the interaction and successive activation of the MRGPRX2 receptor. To further gain insight into the initial events in interactions, we conversely performed MD simulation on the SP-MRGPRX2 complex by pulling SP from the SP-MRGPRX2 complex, and found that stable h-bondings and salt bridges extended up to a distance of 5 nm (**Supplementary Figure 3**). The extended lifetimes of the h-bonds and the salt bridges between SP and MRGPRX2 suggests some deformation in the structure of the receptor during the pulling (unbinding) process. A flexible segment of the receptor containing Glu164 and Asp184 residues, which take part in the formation of the salt bridges, appears to be pulled with the ligand during the process, and as a result, the residues forming salt-bridge, which also participates in h-bond formations with SP, extend into the direction of the pulling force to participate in the interactions. This deformation could be observed in the trajectory of the pulling simulation, and is demonstrated in **Figures 3A, B**, on two snapshots characterized by the center of mass distances of 3.2 and 5 nm. The segment of the receptor of approximately 20 residues (from 164 to 184) extends into the direction of the retreating ligand while being pulled by the salt bridge and h-bonds. The strong interactions with the extending part of the receptor together with a number of h-bonds formed with this part during the unbinding process, conversely, is also suggestive of the binding reaction, which might occur in the exactly reverse order. Examination of the tentative “unbinding” reaction path of SP-MRGPRX2 complex predicted by the pulling simulations suggests that the reverse binding reaction pathway might involve initial salt-bridge and h-bonding interactions with the MRGPRX2 segment containing Glu164 and Asp184, and later gradual “absorption” of the ligand into the binding pocket of the receptor through the increasing number of h-bonds and the salt bridges formed in the pocket.

A generalized motif of peptide ligands has been suggested (16, 17), and a common feature is a well-ordered positioning of basic and hydrophobic residues. The common feature of amino acids has been found to be the likes of hydrophobic residues separated by a group of basic and uncharged residues (17). SP with amino acid sequence Arg-*Pro-Lys-Pro-Gln-Gln-Phe*-Phe-Gly-Leu-Met, clearly bears the motif (italicized residues) and Lys3 forms a part of it. Our results support the hypothesis and further extend on the mechanism of interaction of such motifs which will be governed by h-bonds and salt bridges. Apart from peptide ligands, researchers have also presented the cryogenic electron microscopic structure of MRGPRX2 receptor (14, 15). The ligand binding pocket of MRGPRX2 contains two sub pockets, an electronegative sub pocket consisting of Asp184 and Glu164, and a hydrophobic pocket where Trp243 and Phe170 plays crucial role, and it has been shown that mutations at these sites either inhibit or significantly reduce MGPRX2 activation. With respect to agonists, it has been shown that a peptide agonist having basic characteristics are most likely able to activate MRGPRX2 due to its binding to the electronegative sub pocket, irrespective of their overall conformation (15). These studies support our findings where we show that SP basic residues (Arg1 and Lys3) forms stable interactions with the Asp184 and Glu164 due to h-bond and salt bridges.

To test our computational predictions, we used SP analogs with amino acid substitutions in relevant and non-relevant sites to determine whether they could still activate MRGPRX2 on LAD2. SP derived peptides (SP1, 2 and 4), like SP, caused a rapid decrease in the MRGPRX2 expression on LAD2, suggesting MRGPRX2 mediated mode of cell activation. Further, we studied cell degranulation to examine the potency of peptides in MRGPRX2 mediated LAD2 activation. The results were in accordance with computation findings where removal of Lys3 (SP1 and SP2) limited the activity of analogs, while modifications at the hydrophobic residues (though the overall hydrophobicity was not lost) showed no significant effect on degranulation (SP3-5). In contrast to degranulation (β-hex), the ability of these analogues to activate transcription-dependent mediator production was unexpected. Even though SP1 and SP2 failed to cause β-hex release, there was a considerable amount of MCP-1/CCL2 released by these peptides. Surprisingly, all SP analogs were unable to produce the same amount of TNF as SP, except SP3 and SP5, though at higher concentration (10 µM). These results suggest that subtle changes in ligand structure could alter the binding of ligand to MRGPRX2 and therefore, could induce different signaling cascades, thereby leading to differential mediator production. Thus, it is important to measure both degranulation and *de novo* synthesized mediator production by MC when analyzing MRGPRX2 activation. Finally, we studied the effectiveness of SP1 and SP2 in acting as competitive agonists/antagonists for SP activation of LAD2. SP2 efficiently reduced the amount of β-hex and histamine released from SP-activated LAD2 cells in time dependent manner. Furthermore, peptides SP1, SP2 and SP4 also reduced the level of *de novo* synthesized TNF release upon activation by SP, though no effect was observed for CCL2 release or the release of preformed TNF. These are crucial findings, which suggest that the modulation of the crucial amino acids in a known peptide ligand of MRGPRX2 could help in designing therapeutics for the MRGPRX2 mediated inflammatory disease. Several recent studies have focused on identifying ways to inhibit or block the activation of mast cells through this receptor. In this regard, peptide QWF has been identified which have shown to inhibit MRGPRX2 activation through SP and compound 48/80 (43). Other molecules, which have been identified as an antagonist to MRGPRX2 are Osthole (against SP and compound 48/80), Quercetin (against SP and compound 48/80), Shikonin (against compound 48/80), Saikosaponin A (against compound 48/80), Resveratrol (against compound 48/80), and Roxithromycin (against compound 48/80) (44–50).

## Conclusion

In conclusion, we have shown that SP-MRGPRX2 interaction is mediated by h-bonds and salt bridges. Arg1 and Lys3 in SP were deemed important for SP interaction with MRGPRX2, and that the mutations at key residues within SP changes the activity of the peptide against MCs. SP activated mast cells undergo rapid MRGPRX2 sensitization. Finally, we show that change in the physiochemical properties in a ligand could greatly vary the types and levels of mediators released from activated MCs, which has great significance in the study of disease pathogenesis. Finally, we show that the modulation of the amino acid residue of a known ligand could help in designing MRGPRX2 antagonists.

## Data availability statement

The original contributions presented in the study are included in the article/**Supplementary Material**. Further inquiries can be directed to the corresponding author.

## Author contributions

SH performed the molecular modeling analysis. NA performed the substance P activation studies. SR compiled and analyzed the data and wrote the manuscript. AK provided editorial feedback. MK designed the study, obtained necessary funding, obtained biosafety approvals and assisted in writing the final draft of the manuscript. All authors approved submission of the final version of the manuscript.

## Supplementary material

Supplementary Figure 1 SP-MRGPRX2 Complex 1 **(A)** and Complex 9 **(B)**, as predicted by the SMINA fork of the Autodock Vina docking software. The protein receptor molecules are represented by cartoons with different colors depending on the secondary structure of the residues (white: coil, cyan: turn, magenta: alpha-helix). Ligand molecules are presented as a red cartoon

Supplementary Figure 2 Free energy profiles of the peptides as a function of the center of mass distances between the receptor and the peptide obtained with umbrella sampling simulations for the studied complexes

Supplementary Figure 3
**(A, B)** presents the numbers of h-bonds for every complex depending on the center of mass distance between SP and MRGPRX2 sampled during the pulling simulation of 1 ns; **(C)** presents the distance between the charged residues of the salt bridges formed between SP and MRGPRX2 in Complex 1 (black and red symbols) and 9 (yellow and orange symbols) during 1 ns pulling simulations. Thin horizontal black line denotes the threshold of 0.4 nm below which the salt bridges are assumed to be active.

Supplementary Table 1 Amino acid sequences of SP analogs.
