# Supplementary material for: Substance P analogs devoid of key residues fail to activate human mast cells via MRGPRX2
Source: Front Immunol. 2023 May 9;14:1155740. doi: 10.3389/fimmu.2023.1155740 (PMC10203606; doi:10.3389/fimmu.2023.1155740)
Supplement: Supplementary Figure 1 — SP-MRGPRX2 Complex 1 (A) and Complex 9 (B), as predicted by the SMINA fork of the Autodock Vina docking software. The protein receptor molecules are represented by cartoons with different colors depending on the secondary structure of the residues (white: coil, cyan: turn, magenta: alpha-helix). Ligand molecules are presented as a red cartoon [file DataSheet_1.pdf]

Substance P analogs devoid of key residues fail to activate human mast cells via MRGPRX2

Shammy Raj[1], Stepan Hlushak[2], Narcy Arizmendi[1], Andriy Kovalenko[1], Marianna Kulka[1,3]

[1]Nanotechnology Research Centre
11421 Saskatchewan Drive
National Research Council Canada
Edmonton, Alberta, Canada T6G 2M9

[2]Department of Mechanical Engineering
University of Alberta
Edmonton, Alberta, Canada T6G 1H9

[3]Department of Medical Microbiology and Immunology
6-020 Katz Group Centre
University of Alberta
Edmonton, Alberta, Canada T6G 2E1

Running title: MRGPRX2 activation of human mast cells by substance P requires key residues

Supplementary Information

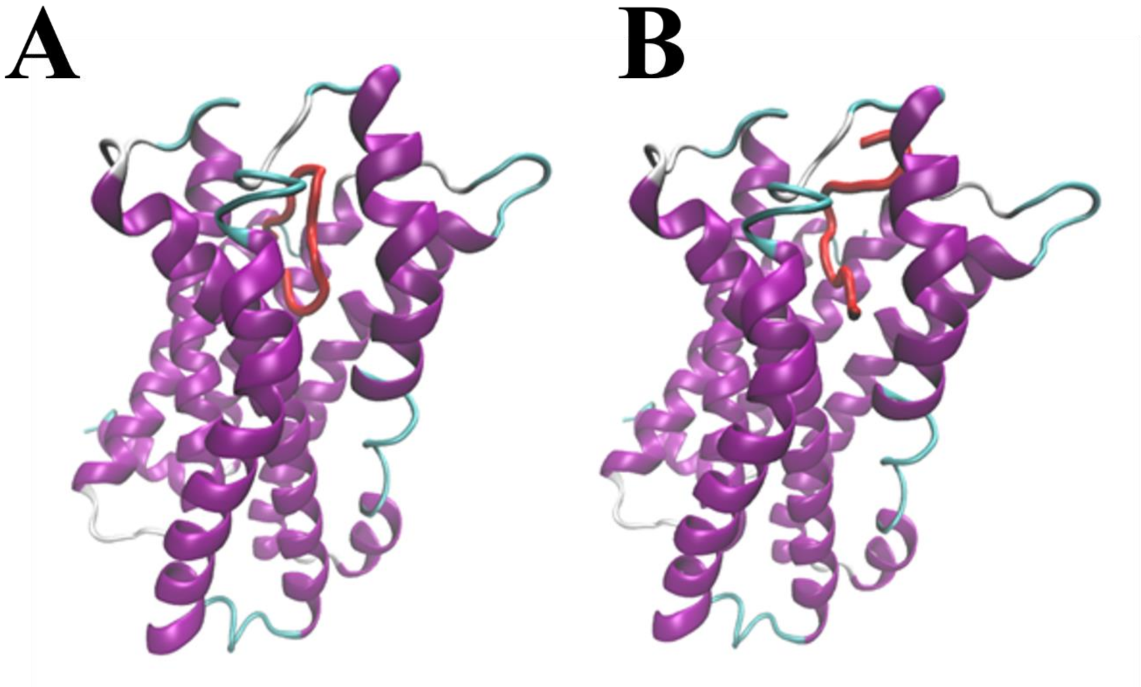

Supplementary Figure 1. SP-MRGPRX2 Complexe 1 (A) and Complex 9 (B), as predicted by the SMINA fork of the Autodock Vina docking software. The protein receptor molecules are represented by cartoons with different colors depending on the secondary structure of the residues (white: coil, cyan: turn, magenta: alpha-helix). Ligand molecules are presented as a red cartoon

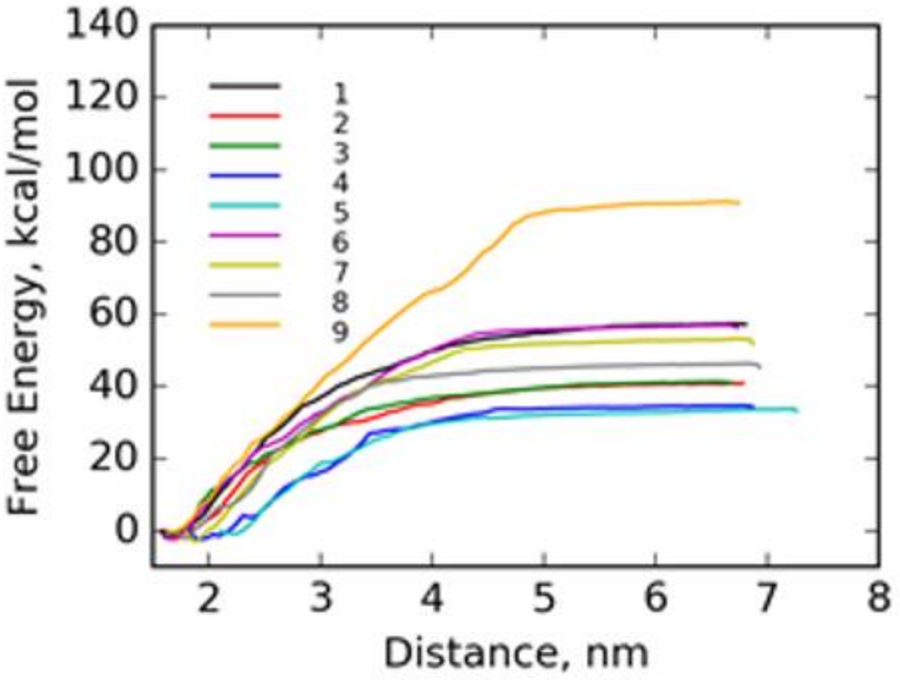

Supplementary Figure 2. Free energy profiles of the peptides as a function of the center of mass distances between the receptor and the peptide obtained with umbrella sampling simulations for the studied complexes

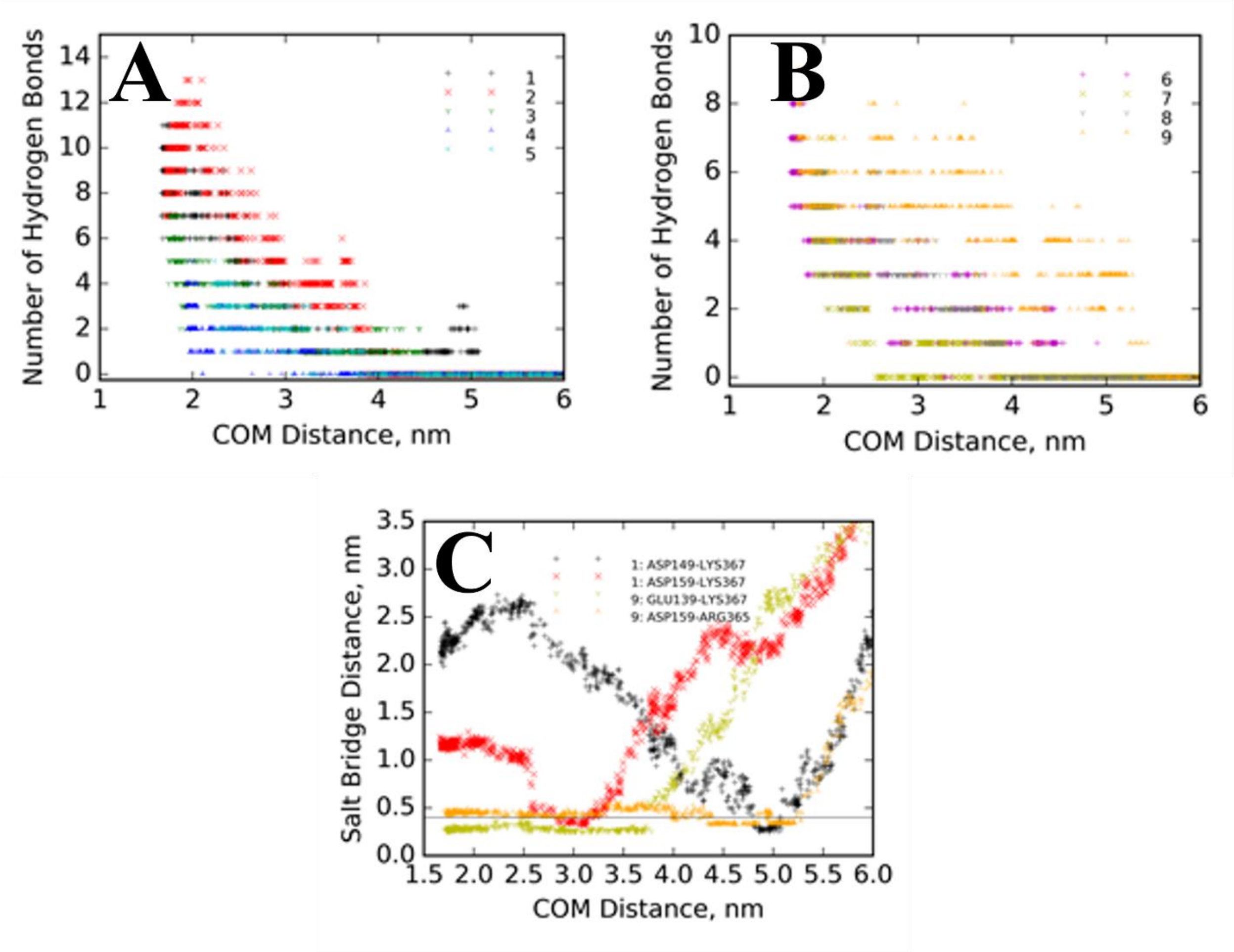

Supplementary Figure 3. (A, B) presents the numbers of h-bonds for every complex depending on the center of mass distance between SP and MRGPRX2 sampled during the pulling simulation of 1 ns; (C) presents the distance between the charged residues of the salt bridges formed between SP and MRGPRX2 in Complex 1 (black and red symbols) and 9 (yellow and orange symbols) during 1 ns pulling simulations. Thin horizontal black line denotes the threshold of 0.4 nm below which the salt bridges are assumed to be active.

Supplementary Table 1. Amino acid sequences of SP analogs

Peptide	Sequence
SP	Arg-Pro-Lys-Pro-Gln-Gln-Phe-Phe-Gly-Leu-Met
SP1	Arg-Pro-Cys-Pro-Gln-Cys-Phe-Tyr-Gly-Pro-Met, disulphide Cys3-Cys6
SP2	Arg-Pro-Cys-Pro-Gln-Cys-Phe-Tyr-Pro-Leu-Met, disulphide Cys3-Cys6
SP3	Arg-Pro-Lys-Pro-Gln-Gln-Phe-Phe-Pro-Leu-Met
SP4	Arg-Pro-Lys-Pro-Gln-Gln-Phe-Phe(4-Bz)-Gly-Leu-Met
SP5	Arg-Pro-Lys-Pro-Gln-Gln-(4-Chloro-Phe)-(4-Chloro-Phe)-Gly-Leu-Met
